# Effectiveness of a screening tool to assess prevention and rehabilitation needs of 45 to 59 years old in primary care – study protocol of a pragmatic randomized controlled trial (PReHa45)

**DOI:** 10.1186/s12913-023-09392-w

**Published:** 2023-04-20

**Authors:** Jennifer Marie Burchardi, Karla Spyra, Martin Brünger

**Affiliations:** Charité – Universitätsmedizin Berlin, corporate member of Freie Universität Berlin and Humboldt-Universität zu Berlin, Institute of Medical Sociology and Rehabilitation Science, Charitéplatz 1, 10117 Berlin, Germany

**Keywords:** Preventive health services, Rehabilitation, General practitioners, General practice, Primary health care, Screening, Access barriers, Randomized controlled trial

## Abstract

**Background:**

For years it has been stated that the need for prevention and rehabilitation is not always identified early enough. Although many individuals have regular contact with a general practitioner (GP), this access path for applying for a prevention or rehabilitation service has not been fully exploited. The important role of GPs in supporting the intention to apply is highlighted in the research. This study aims to evaluate the effectiveness of the “check-up 45 + ” to support GPs both in identifying the need for prevention and rehabilitation services and in submitting applications.

**Methods:**

The study is designed as a two-arm, pragmatic 1:1 randomised controlled study (RCT), which will be conducted in about 20 general practices in the German states of Berlin and Brandenburg. Patients (*n* = 1,654) aged from 45 to 59 years will be recruited by medical assistants. In addition to usual care, both study groups will receive a questionnaire covering socio-economic and occupational variables to be filled out immediately in the waiting room. The intervention group passes through the “check-up 45 + ”. This includes the completion of the “screening 45 + ” that aims to assess the need for prevention and rehabilitation services. Medical assistants will immediately evaluate this 2-page screening tool. If a need is identified and confirmed by the GP, information and application documents will be handed over. Moreover, the application process for rehabilitation services is simplified.

Primary outcome is the proportion of applications for prevention or rehabilitation services financed by the German Pension Insurance. Administrative data will be provided for this purpose. Secondary outcomes include the proportion of approved applications and completed services. In addition, the proportion of persons with a need for prevention or rehabilitation according to the “check-up 45 + ” will be examined. Semi-structured interviews will be conducted and content-analysed to determine the practicability and acceptance of the “check-up 45 + ” by the relevant stakeholders.

**Discussion:**

Prevention and rehabilitation need is insufficiently identified and addressed so far. This study will determine the effectiveness of the “check-up 45 + ” in primary care.

**Trial registration:**

German Clinical Trials Register (DRKS00028303, 03.03.2022).

**Supplementary Information:**

The online version contains supplementary material available at 10.1186/s12913-023-09392-w.

## Background

### Maintaining employability

Due to later retirement age and the increasing lack of qualified personnel, the importance of maintaining or restoring the work ability of older employees is increasing [1, 2]. In order to prevent a reduction in work ability at an early stage and to simplify access to prevention and rehabilitation services, the principle of "prevention before rehabilitation before pension" must be strengthened. In fact, half of those who receive a disability pension for the first time have not received any medical rehabilitation or other services offered by the German Pension Insurance to enhance work ability [3-6]. If a disability pension is granted, employees are very unlikely to return to work [7].

### Needs, application procedure and barriers

A central function of the German Pension Insurance is to promote and maintain the work ability of insured persons by financing rehabilitation measures in specialized rehabilitation facilities with a usual duration of three to four weeks. Furthermore, the German Pension Insurance is to provide prevention services for insured persons with initial health impairments. These prevention services include about three to five full-time days in specialized health care provision (usually rehabilitation facilities) and three to six months extra-occupational group services. Both prevention and rehabilitation services require an application by the insured person to the German Pension Insurance. The prevention application can be filled out online by the patient in a few minutes. The application for rehabilitation is more time-consuming and includes extensive application documents that must be filled out by the patient and physician. Within the study, the application procedure will be simplified by providing the necessary forms, reducing the number of forms required and replacing the doctor’s medical report with a short form.

The German Federal law to strengthen prevention and rehabilitation in working life (*Flexirentengesetz*) provides, according to § 14 (3) of the Social Security Code VI, that for insured persons aged 45 and over, the "introduction of voluntary, individual, work-related health care for insured persons […] is to be tested in model projects". The study presented here is based on this law and is one of about ten model projects in Germany. In addition, the German Federal law to strengthen the participation and self-determination of persons with disabilities (*Bundesteilhabegesetz*) provides that the need for rehabilitation of insured persons is to be recognized at an early stage by the rehabilitation providers using appropriate instruments. So far, there is no active screening to identify a possible need for prevention or rehabilitation. For years, it has been stated that prevention and rehabilitation needs have not always been identified in time and that rehabilitation services should be applied earlier to prevent or mitigate the progression of chronic diseases and disabilities. The application behaviour differs from the subjective and objective need for medical rehabilitation. It is assumed that the need is significantly higher than the number of applications [8-11]. Challenges and barriers for applying are reported both by patients and physicians [12]. In addition to personal, familial, or professional reasons, patients' lack of knowledge about the application process is an obstacle [13-15]. Patients have a great need for support and information on how to apply [9, 13, 14]. Likewise, physicians report information deficits regarding the need for and application of rehabilitation services, and non-transparent refusal criteria [16-18]. There is a need to systematically identify prevention and rehabilitation needs, proactively inform insured persons about services and remove barriers to accessing medical rehabilitation [4, 19].

### Evaluation of the “check-up 45 +” in primary care

Access through the GP practice to apply for prevention or rehabilitation services is still under-used [17, 20, 21]. The important role of GP support as a determinant of intention to apply is highlighted in the research [10, 12, 22-24]. 74% of respondents name their general practitioner or specialist as the "first point of contact for obtaining information" if there is a need for medical rehabilitation [25]. Therefore, it seems useful to improve GPs' ability to recognize the need for prevention and rehabilitation and to facilitate the application process [20, 21, 23, 26].

A range of studies developed and evaluated screening instruments for needs assessment [20, 27-35]. The German Pension Insurance evaluates different approaches in various settings to identify prevention and rehabilitation needs within the framework of the so-called "check-up 45 + " in several model projects. The aim is to establish routine screening approaches nationwide to recognize work-related disability at an early stage, to offer the necessary prevention or rehabilitation services, and to maintain health and employability [36]. In this study, primary care in GP practices is the place where a screening for prevention and rehabilitation needs based on the so-called "screening 45 + " is applied [37]. The "screening 45 + " assesses the five dimensions work ability, mental well-being, functional ability, coping behaviour, and physical activity.

The following research question will be explored: can the number of applications for prevention and rehabilitation services be increased through the “check-up 45 + ” in GP practices?

### Objectives

The aim of this study is to evaluate the effectiveness of the "check-up 45 + ". We will examine whether the "check-up 45 + " affects the number of applications for German Pension Insurance services through an early identification of needs of patients in primary care by a structured needs assessment, provision of information about German Pension Insurance services and a simplified application procedure.

In addition, the practicability and acceptance of the implementation of the “check-up 45 + ” will be assessed among the stakeholders involved.

### Trial design

The “PReHa45” study is a two-arm, pragmatic, 1:1 randomised controlled intervention study (RCT). In GP practices in the German states of Berlin and Brandenburg, patients aged 45–59 years are randomly assigned to an intervention or control group by the practice staff. The intervention group undergoes the "check-up 45 + " in addition to usual care in GP practices, while the control group only receives usual care and completes a general questionnaire on socio-economic and occupational characteristics. The primary criterion for assessing the effectiveness of the "check-up 45 + " is the proportion of applications for prevention and rehabilitation services, determined by administrative data from the individual pension insurance account of the study participants. Practicability, acceptance, and satisfaction with the "check-up 45 + " are examined with semi-structured interviews. The GP practices receive financial compensation for each participant, depending on the effort required. Table 1 shows that according to the nine domains of the PRECIS-2 tool the RCT has a pragmatic approach [38].

**Table 1:** PRECIS-2 domain (1 = very explanatory, 5 = very pragmatic)

	**Domain**	**Score**	**Rationale**
1	**Eligibility Criteria**	4	Inclusion and exclusion criteria (especially age and insurance law requirements) largely correspond to legal requirements and the conditions in usual care (exception: patients insured with other GPI agencies, presumably < 5%; maximum age up to standard retirement age would be feasible; sufficient knowledge of German)
2	**Recruitment Path**	5	Recruitment of study participants in the context of usual care visits to about 20 GP practices; possible selection bias because certain patient groups are not addressed (e.g. in acute consultations, but this would presumably also occur in usual care and would therefore not represent a deduction) or certain people tend to refuse participation (e.g. low social status, language barriers, but this would presumably also be comparable in usual care); easy recruitment, but lack of time and staff and high incidence periods of respiratory diseases in winter leads to stagnation in recruitment
3	**Setting**	5	Identical setting to usual care setting: primary care, where patients usually go for advice and treatment; about 20 GP practices in Berlin and Brandenburg (Germany)
4	**Organisation intervention**	4	Is integrated into usual practice procedures with regular staff with usual knowledge/experience and there is no change in usual care apart from intervention-related ones. However, training is provided on evaluating the "screening 45 + " and on GPI rehabilitation and, above all, prevention services, which were probably not known beforehand
5	**Flexibility of experimental intervention—Delivery**	4	Practices receive a schedule. Concrete implementation of the intervention is flexible and can be adapted to the individual practice procedures. The result of the "screening 45 + " is not mandatory, practices can deviate from it. Other usual care is not influenced by the study; participation in other parallel studies is also possible for patients and practices
6	**Flexibility of experimental intervention—Adherence**	5	Patients are approached in the usual way, the study/participation can be cancelled at any time, the intervention is very short and requires little effort from the participants, therefore adherence is easy to maintain
7	**Follow up**	5	No follow-up
8	**Outcome**	5	Outcome is important for the patient; if there is a need for prevention or rehabilitation, a corresponding therapeutic address in the form of a prevention or rehabilitation service is very relevant for the patient
9	**Analysis**	5	ITT no matter whether compliance

*GP* General Practitioner, *GPI* German Pension Insurance, *ITT* Intention-to-treat, *PRECIS* PRagmatic Explanatory Continuum Indicator Summary

**Fig. 1:**
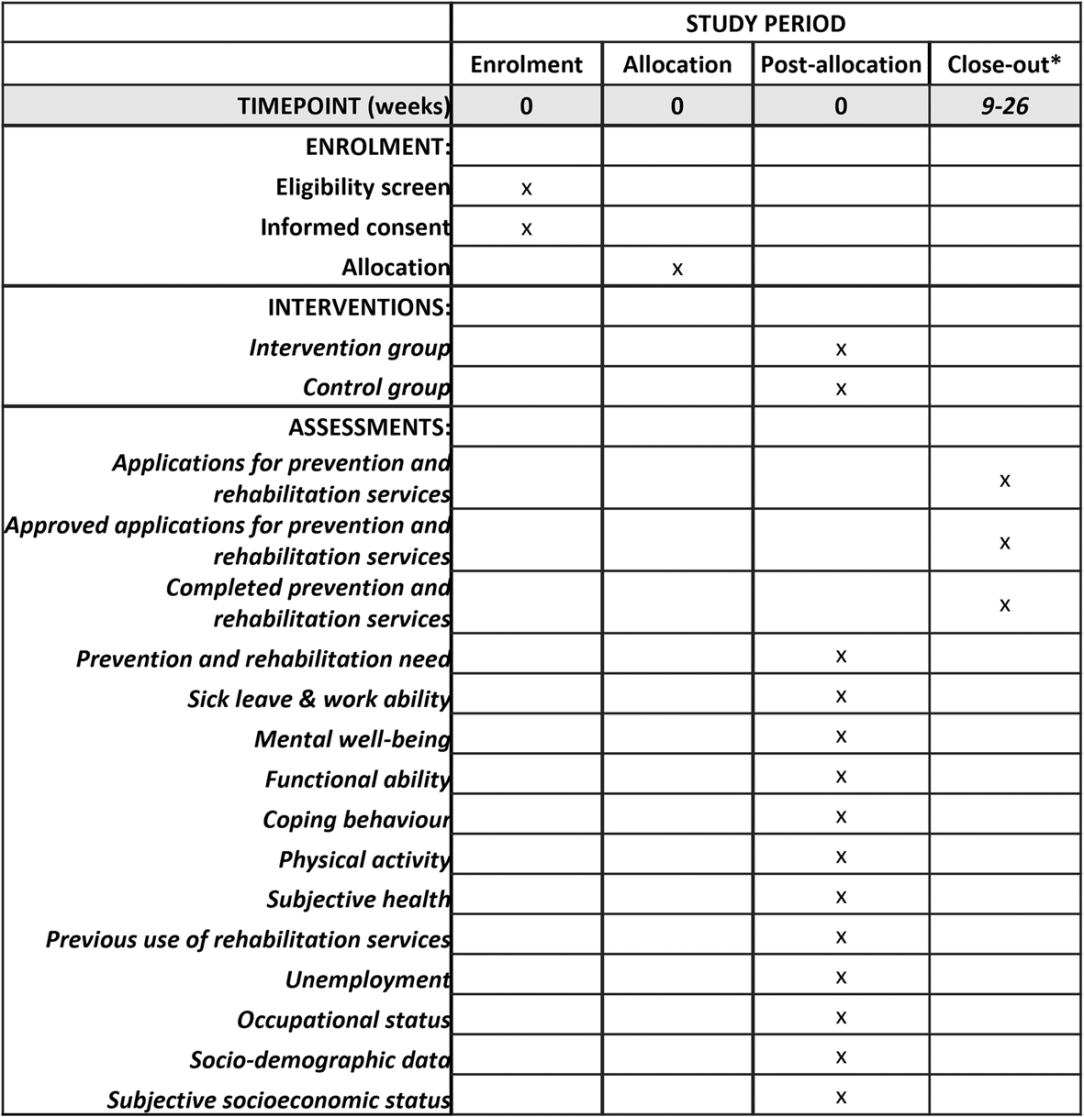
SPIRIT flow diagram of the study

The study protocol is reported according to the Standard Protocol Items: Recommendations for Interventional Trials (SPIRIT) flow diagram (Fig. 1) and checklist (Additional File 1) [39].

## Methods

### Study setting

The intervention is implemented in about 20 GP practices located in Berlin and Brandenburg. Practices in rural and urban areas, each with differing social structures, participate. The practices can have different specialisations (e.g. occupational medicine, naturopathy), but are all maintained by general practitioners or internists practising general medicine. In most practices, one physician and two medical assistants conduct the study; in some practices more persons are involved.

### Eligibility criteria

All patients aged between 45 and 59 years and insured by the German Pension Insurance Berlin-Brandenburg or the German Federal Pension Insurance can participate in the study. Further inclusion criteria are residence in Berlin or Brandenburg, contribution to social insurance for at least 6 months within the last 24 months and sufficient knowledge of German. Excluded are patients who are currently applying for or receiving a prevention or rehabilitation service from the German Pension Insurance as well as those who receive a disability or old-age pension.

### Treatment

#### Intervention

Participants in the intervention group undergo the “check-up 45 + ”, which includes the completion of the "screening 45 + ". This two-page questionnaire is an assessment to identify existing prevention and rehabilitation needs and measures the five dimensions of work ability, mental well-being, functional ability, coping behaviour and physical activity. These dimensions contain partly adapted questions from established instruments that have been evaluated as useful in practice to predict a risk of work disability or to identify existing limitations in the work ability [37]. It will be handed out with an additional two-page questionnaire covering socio-economic and occupational variables by the practice staff, to be filled out in the waiting room. Immediately afterwards, the "screening 45 + " is evaluated by the practice staff by calculating dimension-specific sum scores and applying predefined thresholds in order to assess a potential prevention or rehabilitation need. Each dimension has a value range from 0 to 12. Depending on the score, there are three possible recommendations: (1) no need for a prevention or rehabilitation service, (2) need for a prevention service, and (3) need for a rehabilitation service, as presented in Table 2. If the practice does not agree with the result (e.g. because of its own previous experience with the patient), the practice is free not to follow the screenings-based recommendation and to give another of the three possible recommendations. Based on the final decision, the following further steps of the “check-up 45 + ” result:

**Table 2:** Evaluation of the "screening 45 + "

**Dimension**	**Range**	**Sum score**		**Recommendation**
**Dimension A** *work ability*	0–12	7–12	** → **	Rehabilitation service
		4–6	** → **	Prevention service
		0–3	** → **	No GPI service required
**Dimension B ***mental well-being*	0–12	7–12	** → **	Prevention service
		0–6	** → **	No GPI service required
**Dimension C ***functional ability*	0–12	7–12	** → **	Prevention service
		0–6	** → **	No GPI service required
**Dimension D ***coping behaviour*	0–12	7–12	** → **	Individual measures
		0–6	** → **	No GPI service required
**Dimension E ***physical activity*	0–12	7–12	** → **	Individual measures
		0–6	** → **	No GPI service required

*GPI* German Pension Insurance

(1) If no need for a German Pension Insurance service has been defined, the patient receives a written overview about the result of the "check-up 45 + ". No information material or application forms for German Pension Insurance services are handed out. In case of a high sum score in dimension D or E, the patient may be advised to consider individual health measures. (2) If a need for a prevention service has been defined, the patient receives information material for the prevention services of the German Pension Insurance (RV Fit and DO IT YOURSELF / ONLINE). If desired the patient may apply autonomously for a prevention service. (3) If a rehabilitation service has been defined, patients are supported and guided in the application procedure by the practice staff. They receive information material and all necessary rehabilitation application forms as well as an extract of pre-existing relevant medical findings. The application process is also facilitated for physicians, who do not have to submit the usual medical report form of the German Pension Insurance.

#### Control

The control group is recruited in the same way as the intervention group but is only handed a two-page questionnaire covering socio-economic and occupational variables.

### Outcomes and other measures

Primary and secondary outcomes are recorded using the administrative data (individual pension insurance accounts) from the German Pension Insurance Berlin-Brandenburg and German Federal Pension Insurance and the “screening 45 + ”. A complete list of all measured constructs, sources, value range and scaling is presented in Table 3.

**Table 3:** Source and reference, total score and scaling in the randomized controlled trial

*Outcome*	*Source and reference*	*Total score*	*Scaling*	*Intervention group*	*Control group*
**Primary outcome**					
Applications for prevention and rehabilitation services	GPI registers		Binary	x	x
**Secondary outcome**					
Approved applications for prevention and rehabilitation services	GPI registers		Binary	x	x
Completed prevention and rehabilitation services	GPI registers		Binary	x	x
Persons needing prevention and rehabilitation ^*^	Result of "screening 45 + "	0 to 2	Ordinal	x	
Practicability, acceptance of and satisfaction with "check-up 45 + "	Semi-structured interviews				
**Other measures**					
Sick leave in the past 12 months (Dim. A)^*^	Item from SIMBO [40]	0 to 4	Ordinal	x	x
Expected future work ability (Dim. A)^*^	Adapted item from SIMBO [40]	0 to 3	Ordinal	x	
Self-rated work ability (Dim. A) ^*^	Adapted item from WAI [41]	0 to 5	Ordinal	x	
Mental well-being (Dim. B)^*^	PHQ-4 [42]	0 to 12	Metric	x	
Functional ability (Dim. C)^*^	Adapted items from IRES-3 [43]	0 to 12	Metric	x	x
Coping behaviour (Dim. D)^*^	GPI [37]	0 to 12	Metric	x	
Physical activity (Dim. E)^*^	Adapted items from GPPAQ [44]	0 to 12	Metric	x	
Subjective health status	MEHM/GEDA [45]	0 to 4	Ordinal	x	x
Previous use of rehabilitation services	Own development		Binary	x	x
Current work ability compared with the lifetime best	WAS [46]	0 to 10	Metric	x	x
Subjective prognosis of work ability	SPE [27]	0 to 3	Ordinal	x	x
Unemployment in months	GPI registers		Metric	x	x
Occupational status	GPI registers		Nominal	x	x
Socio-demographic data (gender, age, primary language spoken in household, educational and professional qualifications)	Own development		Nominal/ ordinal/metric	x	x
Subjective socioeconomic status	MacArthur [47]	0 to 10	Metric	x	x

*GEDA* German Health Update, *GPI* General Pension Insurance, *GPPAQ* General Practice Physical Activity Questionnaire, *IRES-3* Indicator of Rehab Status, Version 3, *MEHM* Minimal European Health Module, *PHQ* Patient Health Questionnaire, *SIMBO* Screening Instrument to Assess the Need for Medically and Occupationally Oriented Measures, *SPE* Subjective prognosis of employability, *WAI* Work Ability Index, *WAS* Work Ability Score

^*^Dimension of "screening 45 + "

### Primary outcome

The primary outcome is the proportion of applications for prevention and rehabilitation services of the German Pension Insurance within two months after study participation of each patient.

### Secondary outcomes

Secondary outcomes are (1) the proportion of approved applications for prevention interventions and medical rehabilitation services and (2) the proportion of completed prevention interventions and medical rehabilitation services. In addition, (3) the proportion of persons with a need for prevention and rehabilitation according to the "check-up 45 + " is examined (intervention group only). (4) The practicability, acceptance of and satisfaction with the implementation of the "check-up 45 + " under everyday conditions among participating patients, practice staff and employees of the German Pension Insurance involved in the study will be assessed by semi-structured interviews.

### Other measurements

Health-related and sociodemographic characteristics are assessed via the patient questionnaires and administrative data from the individual pension insurance account. Both groups receive a two-page questionnaire. These variables are collected for sample description, group comparison and to identify relevant covariates related to application for prevention or rehabilitation services. The intervention group additionally completes the two-page”screening 45 + ”.

### “Screening 45 + ”

#### Dimension A—work ability

Work ability is assessed by adapted questions from the "Screening Instrument to Assess the Need for Medically and Occupationally Oriented Measures" (SIMBO) and the "Work Ability Index" (WAI). The SIMBO has already been used in numerous studies [40, 48]. The question on sick leave in the past 12 months with the 5-point scale (0 "not at all" to 4 "more than 6 months") and on the expected future work ability with a 4-point scale (0 "no severe health impairment" to 3 "no longer working at all") were included in a modified form. The WAI is a questionnaire that can provide conclusions about employees' ability to work in relation to their individual conditions and the underlying working conditions [41, 49, 50]. In the "screening 45 + ", the question on the self-rated work ability was adapted with a 6-point scale (0 "no impairment" to 5 "can no longer work at all").

#### Dimension B—mental well-being

Mental well-being is assessed via the short form of the Patient Health Questionnaire-4 (PHQ-4). The PHQ-4 is an ultra-short screening instrument with four items to identify depressive and anxiety symptoms [42]. The items are rated on a 4-point scale from 0 ("not at all") to 3 ("almost every day"). The PHQ-4 is included in the "screening 45 + " in an unmodified form.

#### Dimension C—functional ability

Functional ability is assessed via adapted questions from the generic questionnaire "Indicators of Rehab Status, Version 3" (IRES-3) [43]. Four items were selected from the original scale. In comparison to the original version, the 5-point Likert scale was reduced to four levels for reasons of comparability with the items of the other dimensions. The central answer category was deleted. The level of activity can be documented from 0 ("no problem") to 3 ("impossible").

#### Dimension D—coping behaviour

The German Pension Insurance developed its own instrument consisting of four items to assess coping behaviour [37]. Coping is measured in different domains on a 4-point Likert scale (0 "very good" to 3 "not at all").

#### Dimension E—physical activity

The items on physical activity are adapted from the National Health Service's “German Practice Physical Activity Questionnaire” (GPPAQ). The GPPAQ is a short self-report questionnaire that can be used to measure physical performance [44, 51]. Four items were selected for the "screening 45 + " and translated into German. In each case, the amount of time activity per week is recorded in different areas with four gradations from 0 ("2 h or more") to 3 ("not at all").

### Short questionnaire and administrative data

#### Subjective health status

The subjective health status is assessed, according to a recommendation of the World Health Organization (WHO) [52]. There are five answer options from 0 ("very good") to 4 ("very poor"). This item is part of the Minimum European Health Module (MEHM) [45] and is applied in GEDA – German Health Update [53].

#### Previous use of rehabilitation services

The previous use of rehabilitation services may influence application behaviour [54]; therefore the questionnaire asks whether a rehabilitation has already been carried out.

#### Further measurements of work ability

In the short questionnaire the current work ability compared with the lifetime best is measured by the “Work Ability Score” (WAS) [46], the first item from the WAI. The 11-point scale ranges from 0 ("completely unable to work") to 10 ("maximum ability to work"). The subjective prognosis of work ability is assessed with the SPE scale ("subjective prognosis of employability") [27]. Considering the current state of health and work ability, the survey determines whether the current occupation can be carried out until retirement age (0 “sure” to 4 “definitely not”), whether the general work ability is permanently at risk and whether the person is currently considering applying for a disability pension.

#### Work-related data

The German Pension Insurance provides information on voluntary contributions on days with creditable periods due to unemployment in months and the occupational status.

#### Sociodemographic data

Further data regarding gender, age, educational and professional qualifications, subjective socio-economic status as well as primary language spoken in the household will be assessed via the short questionnaire.

### Sample size estimation

The calculation of the sample size is based on a research project that evaluated the psychometric properties, reliability and criterion validity of the “screening 45 + ” [37]. This study revealed a prevalence of 15.8% in total of persons needing prevention or rehabilitation services among insured persons of the German Pension Insurance aged 45 to 60 (*n* = 4,903): 13.0% showed a need for a prevention service and 2.8% a need for a rehabilitation service. Only a few of the persons with a need for prevention or rehabilitation services intend to submit an application. Moog et al. [11] estimate this proportion at 20%. Thus, we expect 3.2% of the participants in the intervention group to apply for a prevention or rehabilitation service after participation in the "check-up 45 + ". We assume an association between the "check-up 45 + " and the application for a German Pension Insurance service if this takes place within 2 months. For the control group, we calculated the proportion based on the statistics from the German Pension Insurance [55]. Consequently, the proportion of insured persons aged 45 to 59 who submit an application for prevention or medical rehabilitation services within two months is estimated at 0.73%.

**Fig. 2:**
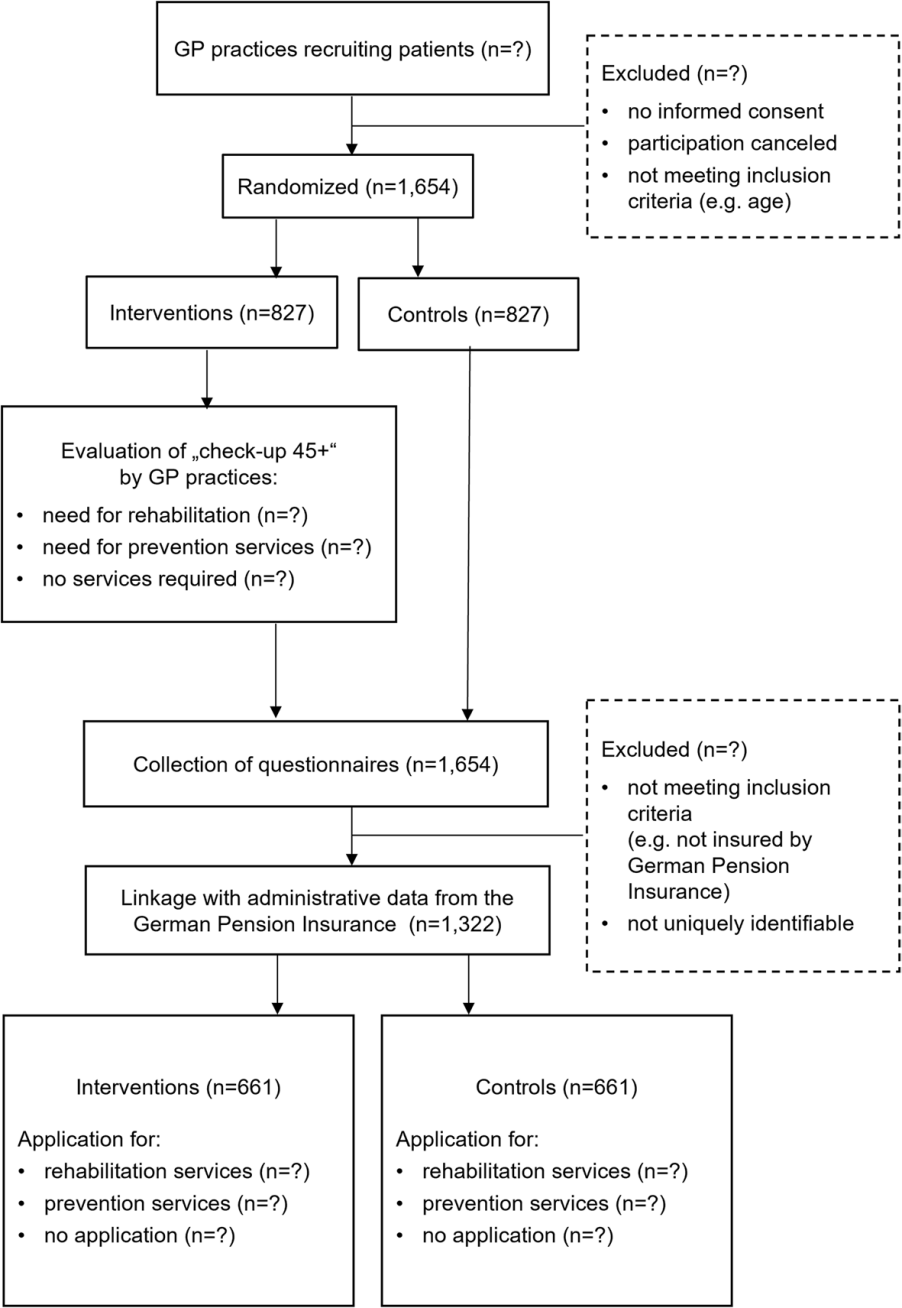
CONSORT flow diagram of the study

In order to detect a difference between the intervention and the control group of applications for prevention and medical rehabilitation services, the power calculation (two-sided test, type I error rate: 5%, power: 85%) resulted in a required minimum sample size of *n* = 661 per group. We assume that a proportion of participants cannot be included into the analyses because they do not meet the inclusion criteria. Some inclusion criteria are difficult for the practice staff to verify when recruiting participants, e.g. insurance status. To take into account a possible drop-out rate of 20%, the targeted sample size is increased to *n* = 827 per group. The Consolidated Standards of Reporting Trials (CONSORT) [56] flow diagram is presented in Fig. 2.

### Recruitment

Before the recruitment phase, the study team visits the GP practices for an one-hour training session on the procedure of the study. The practice staff invite patients who visit the practice for standard care and meet the inclusion criteria (age, insured status) to participate in the study. Study information, consent form and questionnaires are handed out in envelopes for completion. To avoid selection bias, the practice staff is encouraged to address patients who fulfil the inclusion criteria, regardless of their health status and known need for rehabilitation. They should be approached in a neutral way without receiving information about potential prevention or rehabilitation services.

### Allocation

Block randomisation will be performed for each general practice, with the same number of patients per block. Allocation to the intervention or control group is randomised within each block (10 blocks of 10 per practice) to keep the number of case IDs balanced, even if the lists are not finished. The case IDs were generated with R version 4.2.2 and are reproducible. Only the German Pension Insurance is able to link the pseudonyms to the actual persons.

The assignment of the participants included in the study to the intervention or control group is performed randomly, as the practice staff cannot influence the group affiliation of the invited participants during the recruitment process. The practice staff hand out the questionnaires and study documents in sealed, non-transparent envelopes on which the group affiliation is not indicated. The envelopes are only labelled with the questionnaire number (case ID) and are first opened by the participants.

### Blinding

The practice staff are blinded during the recruitment process while inviting patients to the study. Once patients return the completed questionnaires, the practice staff are no longer blinded.

The patients are not aware of their group assignment, as they have no knowledge of the existence of two groups. Unblinding the control group is not intended. Participants in the intervention group are aware of the intervention when they are informed about the result of the "check-up 45 + " after evaluation of the "screening 45 + ". The project coordinators at the German Pension Insurance have no knowledge of the group assignment. The Charité study staff who perform the analyses know the group assignment.

### Data collection

Primary and secondary outcomes are provided by the German Pension Insurance registers and the questionnaire “screening 45 + ” (Tab. 3). Administrative data can be collected reliably and validly for both groups. The German Pension Insurance transmits these data electronically, encrypted and pseudonymised. This enables a complete recording of the administrative data of all study participants. The data collection by patient questionnaires takes place in the GP practices. The study procedure and the questionnaires were tested and adapted in advance in a pre-test with two practices. The practices start at staggered intervals and aim to recruit about 80 participants per practice. Completed questionnaires are stored in the GP practices. During recruitment, Charité study staff regularly visit GP practices to monitor procedures and collect patient questionnaires. Consent forms and study list are sent by the practices to the German Pension Insurance. If participants withdraw their consent, their collected data will be deleted.

After recruitment is completed, semi-structured interviews will be conducted by telephone or in person to investigate practicability and acceptance of the “check-up 45 + ”. Practice staff, participating patients and employees of the German Pension Insurance involved in the study will be interviewed. The interviews are recorded, transcribed, and analysed anonymously.

### Data management

A detailed data protection concept was developed with the data protection officer of the German Pension Insurance Berlin-Brandenburg, which clarifies the rights of participants as well as the organisational procedures for the collection, processing and storage of data. Pseudonymised administrative data of the participants are transferred from the German Pension Insurance Berlin-Brandenburg and German Federal Pension Insurance to the Charité based on the unique case ID for evaluation after completion of the recruitment process.

Collected questionnaires are scanned and verified at the Charité with the data capture system evasys, to assign the pseudonymised questionnaire data to the corresponding case ID and to export them for the analyses. Data entry and data verification are carried out by trained research assistants. The questionnaires and administrative data can be linked via the unique case ID. The electronic data are stored on an internal server of the Charité. Only the study team has access to the data.

### Statistical analysis

All analyses are conducted according to the intention-to-treat principle. Both descriptive and analytical statistics will be used to compare differences between the two study groups. In order to determine the comparability of the two study groups, the distribution of the socio-demographic and health-related variables will be described. Frequencies, means and standard deviations will be calculated according to the scale level. For non-normally distributed data, medians and interquartile ranges will be presented. For the primary and secondary outcomes, absolute and relative frequencies and their 95% confidence intervals will be reported for both study groups. Fisher’s exact test will be applied to test for statistical significance between the intervention and control group. The results will be regarded as significant if the p-value is less than 0.05 (two-sided). Exploratory subgroup analyses will be performed in order to describe differences stratified by gender, age, GP practices and other possible confounders.

The qualitative interview data will be analysed according to the Kuckartz method of qualitative analysis [57].

## Discussion

Needs assessment, information about and claiming of prevention and rehabilitation services to maintain work ability must take place earlier. Many studies are examining interventions to address patients with a prevention or rehabilitation need at an earlier stage. Our study uses a pragmatic approach to investigate the effectiveness and acceptance of a screening tool to assess prevention and rehabilitation needs in primary care. The aim is to examine whether the "check-up 45 + " affects the proportion of applications for German Pension Insurance services through an early identification of needs of patients by a structured needs assessment, by providing information about German Pension Insurance services and a simplified application procedure.

The authors of this protocol will write the final study publications. The use of professional writers is not intended. The findings of our study will be published in articles, conference presentations and in a final report. The study protocol was developed in accordance to the protocol template of SPIRIT (Standard Protocol Items: Recommendations for Interventional Trials) [39].

## Trial status

Recruitment has started and is currently ongoing.

## Supplementary Information

**Additional file 1.** 

**Additional file 2.** 

## Data Availability

Primary data previously used for publications can be made available to researchers in anonymised form at the individual level after a justified and methodologically appropriate request to preha45@charite.de in accordance with data protection regulations for answering the questions formulated in the request. In accordance with data protection regulations, the study data will be retained for a maximum of 10 years after the end of the study.

## Abbreviations

GEDA: German Health Update
GPI: German Pension Insurance
GP: General Practitioner
GPPAQ: General Practice Physical Activity Questionnaire
IRES-3: Indicator of Rehab Status, Version 3
MEHM: Minimum European Health Module
PHQ: Patient Health Questionnaire
SIMBO: Screening Instrument to Assess the Need for Medically and Occupationally Oriented Measures
SPE: Subjective prognosis of employability
WAI: Work Ability Index
WAS: Work Ability Score
WHO: World Health Organization
