# Supplementary material for: Effectiveness of a screening tool to assess prevention and rehabilitation needs of 45 to 59 years old in primary care – study protocol of a pragmatic randomized controlled trial (PReHa45)
Source: BMC Health Serv Res. 2023 Apr 20;23:382. doi: 10.1186/s12913-023-09392-w (PMC10116757; doi:10.1186/s12913-023-09392-w)
Supplement: Supplementary file 2 — Additional file 2. [file 12913_2023_9392_MOESM2_ESM.docx]

**Additional File 1: Items from the World Health Organization Trial Registration Data Set**

| **Data category** | **Information** |
| --- | --- |
| Register | \| German Clinical Trials Register \| \| --- \| |
| Last refreshed on | 14 November 2022 |
| Main ID | DRKS00028303 |
| Date of registration | 03/03/2022 |
| Prospective Registration | Yes |
| Primary sponsor | Charité - Universitätsmedizin Berlin, Institute of Medical Sociology and Rehabilitation Science |
| Public title | Implementation and evaluation of a screening to assess prevention and rehabilitation needs in primary care - A randomised controlled trial |
| Scientific title | Implementation and evaluation of a screening to assess prevention and rehabilitation needs in primary care - A randomised controlled trial - PReHa45 |
| Date of first enrolment | 11/04/2022 |
| Target sample size | 1654 |
| Recruitment status | Recruiting |
| URL | https://drks.de/search/de/trial/DRKS00028303 |
| Study type | Interventional |
| Allocation | Randomized controlled trial |
| Masking | Blinded (patient/subject) |
| Control | Other |
| Assignment | Parallel. Study design |
| Purpose | Screening |
| Phase | N/A |
| Countries of recruitment | Germany |
| Contacts | \| Martin Brünger  Charitéplatz 1, 10117 Berlin, Germany  +49 30 450 517 155  [martin.bruenger@charite.de](mailto:martin.bruenger@charite.de)  Charité - Universitätsmedizin Berlin, Institute of Medical Sociology and Rehabilitation Science  Jennifer Marie Burchardi  Charitéplatz 1, 10117 Berlin, Germany  +49 30 450 517 107  [preha45@charite.de](mailto:preha45@charite.de)  Charité - Universitätsmedizin Berlin, Institute of Medical Sociology and Rehabilitation Science \| \| --- \| |
| Key inclusion and exclusion criteria | Inclusion criteria:  - Insured by the German Pension Insurance Berlin-Brandenburg or the Federation German Pension Insurance  - Contributed to social insurance for at least 6 months within the last 24 months  - Residence in Berlin or Brandenburg  Exclusion criteria:  - Currently applying for or receiving prevention interventions or rehabilitation services from the German Pension Insurance - Currently applying for or receiving an old-age or disability pension - Insufficient knowledge of German  Age minimum: 45 Years Age maximum: 59 Years  Gender: Both, male and female |
| Health conditions or problems studied | Healthy study participants |
| Interventions | Intervention group:  Insured persons of the intervention group receive a short questionnaire to record socio-demographic and occupational characteristics and the “Ü45-Screening” to assess the need for prevention and rehabilitation to fill out in the waiting room of the GP practice. Immediately after completing the questionnaire, the "Ü45-Screening" is evaluated by the practice staff with regard to the need for prevention interventions or rehabilitation services. If a need is identified, education is provided and appropriate information and application documents are handed out to facilitate the application process.  Control group:  Insured persons of the control group receive a short questionnaire to record socio-demographic and occupational characteristics to fill out in the waiting room of the GP practice. |
| Primary outcome | Proportion of applications for prevention interventions and rehabilitation services submitted to the German Pension Insurance Berlin-Brandenburg and the German Pension Insurance Federation within two months after implementation of the intervention |
| Secondary outcomes | 1. Proportion of approved applications for prevention interventions and medical rehabilitation services  2. Proportion of completed prevention interventions and medical rehabilitation services  3. Proportion of persons with a need for prevention and rehabilitation according to the "Ü45-Screening"  4. Practicability, acceptance and satisfaction of the implementation of the "Ü45-Check" under everyday conditions among participating insured persons, practice staff and employees of the socio-medical service of the German Pension Insurance (feasibility study) |
| Secondary ID(s) | \| EA4/215/21 \| \| --- \| \| U1111-1275-0063 \| |
| Source of monetary support | German Pension Insurance Berlin-Brandenburg |
| Ethics review | Status of ethics review: approved  Approval date: 03/11/2021  Contact: (leading) Ethics Committee-No. EA4/215/21 (Ethik-Kommission der Charité -Universitätsmedizin Berlin) |
